# Prevalence of fatigue and consumption of energy drinks consumption among nursing students studying part-time

**DOI:** 10.4102/hsag.v29i0.2487

**Published:** 2024-05-08

**Authors:** Lorato G. Manyeneng, Mogale L. Pilusa

**Affiliations:** 1 Department of Nursing Science, School of Health Care Sciences, Sefako Makgatho Health Sciences University, Pretoria, South Africa; 2 Adelaide Tambo School of Nursing, Faculty of Science, Tshwane University of Technology, Pretoria, South Africa

**Keywords:** energy drink, caffeine, fatigue, nursing, workload, part-time studying

## Abstract

**Background:**

Professional nurses who study part-time are faced with demanding tasks, demanding routine, having to cope with their studies and family commitments. Some nurses try different tactics to cope with their demanding tasks, such as the consumption of energy drinks, to alleviate tiredness and fatigue. Although these energy drinks can alleviate fatigue and boost their energy levels, they have adverse effects to their health such as migraines, insomnia, seizures, arrhythmias and other cardiovascular complications.

**Aim:**

To determine the health effects of energy drinks among nurses studying part-time.

**Setting:**

Selected university in the Gauteng province, South Africa.

**Methods:**

Descriptive, quantitative method that was contextual in nature was used. Self-administered questionnaire was used to collect data from a conveniently sampled population to determine the health effects of the use of energy drinks. Data analysis were done by means of descriptive statistics using the Statistical package for Social Sciences version 26.

**Results:**

Findings indicated that nurses studying part-time experience fatigue (*n* = 86; 49%). To alleviate fatigue (*n* = 91; 52%), they use energy drinks.

**Conclusion:**

Use of energy drinks is prevalent among the nurses because of fatigue caused by studying while working. To reduce the use of energy drinks, the participants need study leave and to be supported by their families and employers.

**Contribution:**

The study encourages reduction or prevent the use of energy drinks by nurses who work and study part-time. Participants must use time management as a coping mechanism.

## Introduction

Studying while working is important as nurses get to improve their qualifications resulting in them being promoted. Nurses work long hours, which requires them to be active and alert while performing cognitively and physically demanding duties, with limited time off between shifts. They experience tiredness and fatigue, which they take long or never recover from, resulting in them resorting to consumption of energy drinks, which are popularly marketed as enhancing energy, and performance. Energy drinks are commonly consumed by those who work long hours, studying at universities or those who need concentration and high levels of physical active (Wassef, Kohansieh & Makaryus 2017:797).

The stressful life of a working student leads them to utilise various methods of overcoming exhaustion and fatigue to be more active and productive. As a result, individuals including working students turn to various products in the market, such as energy drinks (ED). According to Fernandes, Mokwena and Ntuli (2020:2), about 58% of university students use energy drinks to remain awake, be more aware, and help with attention when studying, while 69% of students use alcohol and 16% use alcohol mixed with energy drinks (AmEDs). Working students consume energy drinks trying to alleviate tiredness and fatigue, and that can be more than 500 ml of energy drink per day. Weariness and job stress are generally found to be adversely associated with sleep quality, sleep quantity and perceived health status among nurses (Lin et al. 2014:605).

Because of the nature of their career, working students reported drinking moderate to high quantities of caffeine on a regular basis to counteract excessive fatigue and workplace stress (Higbee et al. 2020:26). Kim and Kim (2015:24) indicated that working nursing students consume energy drinks during their examinations amounting to 1–30 cans per week as a way of fighting fatigue. In addition, nurses consuming ED reported higher levels of perceived stress than non-energy drink consumers (Higbee et al. 2020:24). Energy drinks that are popular in South Africa are Punch energy, Predator energy, MoFaya energy, Score energy, Slayer energy, Red Bull energy, Power Play, Dragon, Switch, ReBoost, Bioplus, Monster, Lucozade and Move. They are sold freely to any individual irrespective of their age.

According to Lin et al. (2014:604), in Taiwan, the majority of nurses (83.3%) who worked night duty and shifts reported poor sleep quality, including trouble falling asleep and the use of sleeping medications. Pavlovic et al. (2023:1) add that 52.4% of students at the University of Osijek (Eastern Croatia) consume ED, and male students were more likely to consume ED than females. Energy drinks contain a variety of ingredients such as Ginkgo biloba, sugars, kola nut and cyanocobalamin (B12), including glucuronolactone (a glucose metabolite). Other additional ingredients in energy drinks are guarana (contains caffeine, teobromin and teofiline), taurine (an amino acid), niacin, pyridoxine and riboflavin (B2). Furthermore, ingredients found in energy drinks such as ginseng extract, inositol (B8), ephedra, yohimbine, theophylline, L-carnitine, vitamins and herbs are addictive. Marczinski et al.’s (2014:57) research has discovered that while energy drinks enhanced subjective assessments of vigour and weariness, objective performance did not improve and appeared to worsen over a period. Sleeping patterns can be affected by the consumption of energy drinks. This was supported by a study done to compare caffeine-only and non-caffeine consumers and nurses who took energy drinks had poorer sleep quality and shorter sleep hours (Higbee et al. 2020:24). While experts and the public have generally ignored the risks of excessive energy drink intake among young people and their long-term effects, they may become a severe public health problem in the future (Yusupova & Firdavs 2022:24). There are few literatures on the health effects of energy drinks as alluded by; hence, a new publication is needed (Yusupova & Firdavs 2022:24).

## Research aim

The purpose of the research was to determine the prevalence of fatigue and consumption of energy drinks among nurses studying part-time at a selected university in Gauteng, South Africa.

## Research methods

### Research design

The study’s research strategy was a descriptive, quantitative method that was contextual in nature. This design was used to collect data to determine the prevalence of fatigue and consumption of energy drinks by working students studying part-time at a selected university in Gauteng, South Africa.

### Setting

The research was carried out at a conveniently selected university in Gauteng, South Africa. At the time of the study, the university’s nursing department was offering basic B. Tech nursing degree, which was offered full-time, a postgraduate B. Tech occupational health nursing programme and a postgraduate B. Tech Oncology nursing programme that were offered on a part-time basis. Programmes are offered on part-time basis, where students attend 1 week every month over a period of 8 months. Students are required to completed five theoretical modules and a practical module consisting of 900 hours prior to registration as occupational health nurse.

### Population

The target population of the study was 375 working students who were studying part-time towards a B. Tech in occupational health nursing.

### Sample and sampling technique

A total of 191 students were conveniently sampled from the target population. The statistician advised that 191 represents more than 50% of the population and will contribute positively to the study. The inclusion criteria for the study were that the respondents must:

1. Be registered at the selected university as a part-time nursing student.
2. Be a B. Tech occupational health nursing student in the first or second year of their study.
3. Be employed for more than 1 year by their employer.

### Pre-testing of the data collection instrument

The tool was pre-tested on 10 working students who met the inclusion criteria but did not participate in the main study. Pre-testing was performed to determine the instrument’s validity and reliability, as well as to determine how well the respondents comprehended the questions on the instrument. It was also done to allow the researcher and field staff to practice presenting the questions to the respondents and also to determine the time required to complete the questionnaire, which was determined to be 30 min.

### Data collection procedures and instruments

An English self-developed questionnaire that had open- and close-ended questions was distributed to the first 191 respondents who were conveniently sampled. The distribution was done at their institution of learning at the time and date agreed upon with the respondents.

Before collecting data, the ethical committee of the university where the study was conducted was consulted, and ethical clearance was obtained. Permission to conduct the research was also acquired from the nursing department where the study’s participants were enrolled for the programme. Before data collection could begin, each respondent was asked to sign an informed consent form. Confidentiality and anonymity were guaranteed to the respondents, as the information gathered did not link to any respondents, and their names did not appear in any document resulting from the study. The respondents were free to leave the research at any time without the risk of repercussions.

Distribution of the self-administered questionnaire was done conveniently to the first 191 sampled respondents when they came to class on their block sessions, to complete and return them to the researcher on an agreed-upon day, which was the next block for attendance. Data collection was done over a period of 1 month to ensure the majority of students will have time to complete the questionnaire, even those who were absent when questionnaires were distributed. After completion of the questionnaire, the researcher collected them on the date and time agreed upon with the respondents. Only 175 (*n* = 92%) questionnaires were used as 11 (*n* = 6%) respondents did not return the questionnaires and five (*n* = 3%) questionnaires were spoiled by the respondents and they were not used for the study.

### Data analysis

The data collected was captured into Microsoft Excel 2010 and then exported to SPSS 26 for data analysis. The analysis of data was done by means of descriptive statistics using version 26 of the Statistical Package for Social Sciences to ensure synthesise and describe the collected data.

### Ethical considerations

Ethical clearance to conduct this study was obtained from the Tshwane University of Technology, Faculty Committee Research Ethics (No. FCRE: 2017/08/001 [SCI] [2]).

## Results

The results discussed in the below section include demographic information and experiences with regard to fatigue and consumption of energy drinks by the respondents.

### Demographic information

The demographic information (see Table 1) includes the marital status and the gender of the respondents. The respondents who took part in the study were 175 and 142 (81%) were females while 33 (19%) were males. Furthermore, among the 175 respondents, 81 respondents (46%) were single, eight respondents (5%) were divorced, 82 respondents (47%) were married, and four respondents (2%) were widowed.

**TABLE 1:** Demographics of the study findings.

Demographics type	Demographic	*N* = 175	%	Types of questions
Gender	Male	33	19	Close-ended questions
	Female	142	81	
Marital status	Single	81	46	Close-ended questions
	Married	82	47	
	Divorced	8	5	
	Widowered	4	2	

As stated in Table 2 of the 91 respondents who consumed energy drinks, 5 (5.5%) respondents consumed ED only once a week, 15 (16%) respondents consumed ED 2–3 times a day, 19 (21%) respondents consumed ED more than three times a day, while 52 (57%) respondents consumed ED once a day. In addition, Table 2 that about 91 respondents (*n* = 91; 52%) consumed energy drinks while studying part-time. As indicated on Table 3, among the 91 respondents (100%) who consumed energy drinks, 57 (63%) stated that their reasons for consuming energy drinks are that it keeps them alert as they turned to be exhausted most of the time, 13 (14%) respondents indicated that energy drinks helped them remain awake when they feel sleepy and 10 (11%) consumed caffeine for pleasure. Furthermore, 11 (12%) respondents indicated that they drink energy drinks because they are addicted to them and cannot do without it (see Table 3). As indicated by Turnbull et al. (2017:169), energy drink consumption predisposes the individual’s total cardiovascular system to cardiac arrest, stroke, coronary heart diseases, arrhythmias, heart failure and hypertension.

**TABLE 2:** Consumption of energy drinks.

Consumption of energy drinks	Number of respondents	%
Once a week	5	5.5
Once a day	52	57
Two to three times a day	15	16
More than three times a day	19	21

Note: Total number of respondent = 91.

**TABLE 3:** Reasons for consuming energy drinks.

Reasons for consumption of energy drink	Number of respondents	%
It keeps them alert or active	57	63
Keeps them awake when they feel sleepy	13	14
Consumed it for pleasure	10	11
They are addicted to energy drinks	11	12

Note: Total number of respondent = 91.

As stated on Table 3 from 91 respondents who consumed energy drinks, 57 (63%) respondents stated that they use it as it kept them alert, 13 (14%) respondents consumed ED as it kept them awake when they felt sleepy, 10 (11%) respondents consumed ED for pleasure, while 11 (12%) respondents consumed ED as they are addicted to drinking it. This is alluded by Fernandes, Mokoena and Ntuli, (2020:2) as stated that about 58% of university students use energy drinks to remain awake, be more aware and help with attention when studying.

## Discussion

The objective of this study was to determine the prevalence of fatigue and consumption of energy drinks by employed professional nurse students studying towards an academic qualification in occupational health nursing. The study results have confirmed the stipulation by Wassef et al. (2017:797) that with increased stress to perform academically, and at work, it is not surprising that most consumers of EDs are teenagers and young adults.

### Energy drinks

Energy drinks or caffeinated energy drinks (CEDs) as known in other countries have been available and consumed in the United States since the late 1940s (Alsunni 2015:469), and it was marketed as ‘Dr. Enuf’ while in Europe, ED arrived only in the late 1980s. According to Lin et al. (2014:604) in Taiwan, the majority of nurses (83.3%) who worked night duty and shifts reported poor sleep quality, including trouble falling asleep and the use of sleeping medications. Pavlovic et al. (2023:1) add that 52.4% of students at the University of Osijek (Eastern Croatia) consumed ED, and male students were more likely to consume ED than females. Alsunni (2015:470) reported a case of acute renal injury in a 40-year-old man after consuming energy drinks on a regular basis for about 2–3 weeks. The research conducted on somewhat sleep-deprived healthy individuals taking energy drinks showed that the impact of consuming energy drinks lasted up to 6 h (Wassef et al. 2017:796). Students can take more energy drinks, especially during their study or exam periods, which mostly exceed 6 h. Marczinski et al.’s (2014:57) stated that research has discovered that while energy drinks enhanced subjective assessments of vigour and weariness, objective performance did not improve and they appeared to worsen over time.

Energy drinks (EDs), which include particular stimulating substances, are becoming more popular in the market (Pavlovic et al. 2023:1), and there are no regulations that restrict individuals especially teenagers to buy ED. Recently, in South Africa, Prime hydration drink was released. Adults, young children and teenagers were in long queues to buy it at different supermarkets. According to Sports Science Institute of South Africa (SSISA), in 2023 the hydration drink contains electrolytes, Vitamin B, 10% coconut water, branch chain amino acids (BCAAs), 2 g of sugar per drink, and many antioxidants. Even though Prime website states that hydration drink is suitable for all ages, other sources have advised children under 15 years not to consume the drink (SSISA 2023). The Prime ED, which is currently unavailable in South Africa, is the most worrisome product as it contains 200 mg of caffeine, which is much higher compared to its competitors such as Monster with 160 mg per can, and Red Bull with only 80 mg per can (SSISA 2023). In addition, the acute consumption of ED in healthy children and teenagers is associated with a significantly increased systolic blood pressure (SBP) and diastolic blood pressure (DBP) (Oberhoffer et al. 2022:8).

Individuals who are in danger of consuming ED are competitive athletes, people with underlying cardiovascular disease, young people, caffeine-naive or caffeine-sensitive people, underlying hypertension, and pregnant women (Higgins, Yarlagadda & Yang 2015:104; Wassef et al. 2017:797). In addition, ED usage is predominantly a health concern among adolescent and young adult males (Oberhoffer et al. 2022:2; Wassef et al. 2017:797), and as it is associated with an increase in substance misuse and risk-taking behaviours (Ali et al. 2015:308).

Higgins and Ortiz (2014:1) state that EDs contain a variety of ingredients such as Ginkgo biloba, sugars, kola nut, cyanocobalamin (B12), glucuronolactone (a glucose metabolite), guarana (contains caffeine, teobromin and teofiline), taurine (an amino acid), niacin, pyridoxine, riboflavin (B2), ginseng extract, inositol (B8), ephedra, yohimbine, theophylline, L-carnitine, vitamins and herbs. Energy drinks that are popular in South Africa are Punch energy, Predator energy, MoFaya energy, Score energy, Slayer energy, Red Bull energy, Power Play, Dragon, Switch, ReBoost, Bioplus, Monster, Lucozade and Move. They are sold freely to any individual irrespective of their age. Fernandes et al. (2020:1) alluded that in South Africa figures showed that between 2009 and 2014, yearly turnover of energy drinks escalated from 97.7 million litres to 167.7 million litres meaning more and more individuals were consuming energy drinks, while according to South African sport and energy drinks market insights report 2022, annual turnover was eighty-five million seven hundred twenty thousand dollars equivalent to One hundred sixty-six billion seven hundred thirty-nine million one hundred fifteen thousand two hundred rands -R1.6 billion. This is evident that energy drinks are highly consumed by most individuals in South Africa.

The intake of energy drinks can result in several health risks to the consumers such as migraines, insomnia, seizures, anxiety, chest pain, agitation, arrhythmias, metabolic acidosis, hallucinations, gastrointestinal upset and other cardiovascular complications. According to Ali et al. (2015:308), the cardiovascular and neurological systems are the most commonly affected after increased substance abuse of ED. When compared to a placebo, energy drinks have been demonstrated to induce hypertension (Wassef et al. 2017:797). There are several cardiovascular complications that can either be acute or chronic effects related to the consumption of energy drinks (Higgins et al. 2015:107). In addition, the high intake of energy drinks has been associated with numerous medical problems such as migraines, insomnia, seizures, hallucinations, gastrointestinal upset, anxiety, chest pain, agitation, arrhythmias, metabolic acidosis and other cardiovascular complications (Higgins et al. 2015:106). Wassef et al. (2017:797) add that globally, caffeine is thought to be connected with a variety of cardiac co-morbidities, including high numbers of arrhythmias and palpitations such as ventricular ectopy, supraventricular and atrial fibrillation.

These complications result because energy drinks contain ingredients that are dangerous to several systems including the neurological and cardiovascular systems (Ali et al. 2015:308). Seizures were the most common neurological consequences although others included neuropsychotic agitation, violent conduct and suicide ideation (Wassef et al. 2017:798). Energy drinks have different ingredients; however, the most common ingredients include sugar, caffeine, taurine, ginseng and guarana (Elitok et al. 2015:920; Grasser et al. 2014:1562). These ingredients have effects on certain systems. As indicated by Wassef et al. (2017:796), throughout the previous decade, the consumption of energy drinks has been viewed with scepticism as potentially dangerous because of their perceived high caffeine concentration, as well as additional ingredients such as taurine, guarana and L-carnitine that are mostly unknown to the general population. According to Wassef et al. (2017:796), other countries including France once prohibited the usage of Red Bull, but the European Union overturned the restriction because of a lack of proof supporting its toxicity to the human body.

### Sugar

Energy drinks often have a high sugar content, ranging from 21 g to 34 g per serving/can. In addition, the majority of the sugar comes in the form of sucrose, glucose or high-fructose corn syrup; hence, excessive consumption of energy drinks may increase the risk of obesity and type 2 diabetes (Alsunni 2015:470). Furthermore, energy drinks’ low pH and high sugar content were linked to a 2.4-fold increase in tooth deterioration or decay. Pinto et al. (2013:1) found that high consumption of energy drinks may cause cervical dentin hypersensitivity by eliminating the smear layer of the teeth.

### Ginseng

As indicated by Palhares et al. (2015:14) and Wassef et al. (2017:802), ginseng is an East Asian (mainly in China and Korea) plant and is the world’s most popular herbal supplement and has also been used as an ingredient in energy drinks. This herb is known to improve memory, cure diabetes, wide-reducing stress, erectile dysfunction, insomnia and increase stamina (Wassef et al. 2017:803). Americans believe taking ginseng will not only provide bodily benefits but will also improve their cognitive performance and well-being (Mishra & Verma 2017:516). The amounts of ginseng found in EDs are thought to be less than the amount needed to deliver the suggested therapeutic benefits or cause adverse events (Wassef et al. 2017:803). According to a 2006 World Health Organization review, ginseng saponins ‘are thought to decrease serum prolactin, thereby increasing libido’ in male impotence. However, excessive consumption of ginseng may cause insomnia, diarrhoea, headache, hypertension, rashes, insomnia, Stevens-Johnson syndrome and irritability (Wassef et al. 2017:803).

### Guarana

According to Wassef et al. (2017:802), guarana is also known as Paullinia cupana from South America, and as early as 1872 it was used for the treatment of ‘Sick-Headache’. According to the Food and Drug Administration (US FDA), guarana is widely regarded as safe, though no dosages have been determined, and it is unclear how much guarana is in each drink because many producers do not give a milligram amount. In addition, it is stated that guarana seeds contain 2% – 4.5% caffeine, whereas coffee beans have 1% – 2% caffeine (Wassef et al. 2017:802). In a can of 450 mL of energy drink, the quantity of guarana ranges from 1.4 mg to as much as 300 mg (Wassef et al. 2017:802).

### Caffeine

The most commonly used ingredient in ED is caffeine, which is extracted from the uncooked or raw fruit of over 60 different coffee plants (*Coffea arabica*), all of which are members of the methylxanthine family (Campbell et al. 2013:2). According to Wassef et al. (2017:797), caffeine’s ability to raise blood pressure quickly is thought to stress the cardiovascular system, increasing the probability of arrhythmia. In addition, in other research, acute caffeine consumption of 200 mg impairs hyperaemic myocardial blood flow response during exercise in patients with coronary artery disease (Grinberg et al. 2022:628). Caffeine found in energy drinks has been demonstrated to increase diuresis (Alsunni 2015:470). Because of the high level of caffeine, it was discovered that compared to when individuals consumed 355 mL can of Red Bull and consumed 300 mL of water, Red Bull imposes a cumulative cardiovascular burden, increasing systolic blood pressure by about 10 mmHg, diastolic blood pressure by about 7 mmHg, heart rate by about 20 beats/min and lowering cerebral blood flow velocity by −7 cm/s (Wassef et al. 2017:797). As indicated by Alsunni (2015:469), caffeine intoxication symptoms usually appear when the intake is equal to or more than 200 mg. Moreover, high caffeine consumption is linked to acute and persistent daily headaches by inducing a pro-nociceptive state of cortical hyperexcitability (Alsunni 2015:469). According to La Vieille et al. (2021:1020), a new health risk assessment confirmed Health Canada’s guideline that healthy adults consume no more than 400 mg of caffeine per day. Consuming ED can be addictive as individual use them to be alert while studying Yusupova and Firdavs (2022:25). According to Yusupova and Firdavs (2022:25), scientists and specialists have differing perspectives regarding ED, some believe that energy drinks are as safe as regular cola, while others believe that they can function like drugs and induce addiction and dependence.

### Limitations of the study

The nature of the study was contextual; therefore, the results cannot be generalised to all working nurses who are studying part-time. Follow-up on responses where further information was required by the researcher was not done as the promotion of anonymity meant that the gathered data could not be linked to respondents.

### Recommendations of the study

The nursing students studying part-time should be given insights about energy drinks and their disadvantages as part of their health education module and good nutrition. Early planning on how they are going to tackle their studies and sticking to the plan is advised.

## Conclusion

Being a student and working can be beneficial in the long term as companies will have employees with enriched knowledge and additional qualifications. The research results indicated that the respondents experienced fatigue and resorted to consuming energy drinks during their studies, which can have a detrimental effect on their health.
